# Cold-inducible RNA binding protein (CIRP), a novel XTcf-3 specific target gene regulates neural development in *Xenopus*

**DOI:** 10.1186/1471-213X-8-77

**Published:** 2008-08-07

**Authors:** Stephanie van Venrooy, Dagmar Fichtner, Martin Kunz, Doris Wedlich, Dietmar Gradl

**Affiliations:** 1 Zoologisches Institut II, Universität Karlsruhe (TH), 76131 Karlsruhe, Germany

## Abstract

**Background:**

As nuclear mediators of wnt/β-catenin signaling, Lef/Tcf transcription factors play important roles in development and disease. Although it is well established, that the four vertebrate Lef/Tcfs have unique functional properties, most studies unite Lef-1, Tcf-1, Tcf-3 and Tcf-4 and reduce their function to uniformly transduce wnt/β-catenin signaling for activating wnt target genes. In order to discriminate target genes regulated by XTcf-3 from those regulated by XTcf-4 or Lef/Tcfs in general, we performed a subtractive screen, using neuralized *Xenopus *animal cap explants.

**Results:**

We identified cold-inducible RNA binding protein (CIRP) as novel XTcf-3 specific target gene. Furthermore, we show that knockdown of XTcf-3 by injection of an antisense morpholino oligonucleotide results in a general broadening of the anterior neural tissue. Depletion of XCIRP by antisense morpholino oligonucleotide injection leads to a reduced stability of mRNA and an enlargement of the anterior neural plate similar to the depletion of XTcf-3.

**Conclusion:**

Distinct steps in neural development are differentially regulated by individual Lef/Tcfs. For proper development of the anterior brain XTcf-3 and the Tcf-subtype specific target XCIRP appear indispensable. Thus, regulation of anterior neural development, at least in part, depends on mRNA stabilization by the novel XTcf-3 target gene XCIRP.

## Background

The most prominent nuclear transducer of wnt/β-catenin signaling in the nucleus belong to the Lef/Tcf family of sequence specific HMG-box transcription factors. Higher vertebrates express four distinct members of this family, Tcf-1 (or Tcf-7), Tcf-3, Tcf-4 (or Tcf-7 like 2) and Lef-1 [1,2]. In *Xenopus*, XTcf-1, XTcf-3 and XTcf-4 are maternally provided, while the expression of XLef-1 starts only after mid-blastula transition [3]. The expression of XTcf-1, XTcf-3 and XLef-1 is widespread and overlapping in many tissues, with highest levels in the head region including brain, head mesoderm and branchial arches [3,4]. Zygotic expression of XTcf-4 is restricted in neurula and tailbud stages to the anterior midbrain [5] and in tadpoles to the entire midbrain [6].

A partial functional redundancy of Lef/Tcf family members has been shown in mice, where the Tcf-1/Lef-1 and Tcf-1/Tcf-4 double knock-out mice revealed more than additive effects compared to the corresponding single knock-out mice [7,8]. In *Xenopus*, however, the individual Lef/Tcfs appear to have more non-redundant roles. Although XTcf-1, XTcf-3 and XTcf-4 are maternally provided, it has been shown by rescue experiments of Lef/Tcf depleted embryos that XTcf-1 mediated target gene activation in partial redundancy with XTcf-4, and XTcf-3 mediated target gene repression are required for establishing of the dorsal embryonic axis [9] and mesoderm patterning [10]. Zygotic XTcf-4 has been shown to regulate midbrain development [6]. XLef-1 is necessary for mesoderm patterning [11]. These non-redundant roles of Lef/Tcfs during early *Xenopus *development implicate that the individual transcription factors regulate a different subset of target genes.

It seems most likely that Lef/Tcfs are general regulators of brain development because their expression overlaps in the developing brain and posteriorization of the developing CNS is regulated by wnt/β-catenin signaling [12]. Thereby, they seem not to play a role as inducing factors, driving the cell fate towards anterior neural development, because they can not induce the expression of neural marker genes in *Xenopus *animal cap explants. Instead, they rather appear to be involved in the stabilizing and transforming step in the model of Stern [13]. However, dorsal injection of a dominant negative Tcf-3 construct resulted in a repression of Bmp4 and subsequently in a downregulation of the pan-neural marker gene nrp1 [14]. This is discussed as β-catenin independent target gene activation by a physical interaction between XTcf-3 and Frodo/Dapper [15].

In order to identify genes specifically regulated by XTcf-3, we subtracted the transcriptome of neuralized animal caps of Tcf-4 morpholino injected embryos from those of Tcf-3 morpholino injected ones and identified among others *Xenopus *cold inducible RNA binding protein (XCIRP) as novel XTcf-3 specific target gene. XCIRP was originally identified in a screen for target genes activated after retinoic acid treatment of activin treated explants [16]. Later it was found in a screen for genes, which are upregulated in animal caps neuralized by smad7 [17]. In *Xenopus *three XCIRP isoforms, XCIRP, XCIRP1 and XCIRP2 have been reported. Because XCIRP and XCIRP1 mRNA is to 98% identical resulting in proteins of 99.4% identity they are referred in here further on as XCIRP. XCIRP protein is to 94% identical to XCIRP2, implicating that these isoforms derive from different alleles of the pseudotetraploid *Xenopus laevis*.

XCIRP is highly expressed in developing oocytes and forms together with FRGY2 and mRNP3 the most abundant group or RNA binding proteins in oocytes [18,19]. It interacts with and is methylated by the protein-arginine methyltransferase 1 (XPRMT1). This methylation is supposed to play an essential role in regulating the subcellular localization of XCIRP [20]. XCIRP interacts with ELAV-like RNA-binding protein (XELrA) in *vitro *and in *vivo *and inhibits deadenylation of synthetic AU rich elements [19]. Immunoprecipitation followed by RT-PCR revealed that many different mRNAs including cyclin B1, CAF1 p150 (Chromatin assembly factor 1 p150 subunit) Nek2B, Cytochrome C oxidase peptide VIb and bystin bind to XCIRP protein in *Xenopus *oocytes [19]. In a similar assay, Peng et al. [21] identified α- and β-catenin as well as C- and E-cadherin mRNA bound to XCIRP protein. Binding of XCIRP to the above mentioned mRNAs appears to increase their stability [21]. Whether binding and stabilization of mRNA holds true only for a small subset of mRNA or if mRNA in general is stabilized by XCIRP remains elusive.

Although almost ubiquitously expressed at high levels during gastrulation [16], first effects of XCIRP knockdown by antisense morpholino injection were not seen before stage 22, where more than half of the embryos died [22]. More specific effects of the XCIRP morpholino were observed only after targeting the injection to the C3 blastomere in 32 cell stage embryos. This resulted in malformation of the pronephros [22].

Here we show by antisense morpholino oligonucleotide injection followed by *in situ *hybridization that the expression of XCIRP depends on XTcf-3 but not on XLef-1 or XTcf-4. The expression fields of XTcf-3 and XCIRP broadly overlap in the neural tissue and both, the XTcf-3 specific and the XCIRP specific anitsense morpholino oligonucleotide lead to a similar phenotype, a broadening of the neuroectoderm. We provide evidence, that a general stabilization of mRNA by the XTcf-3 target gene XCIRP is an essential step in early neural development.

## Results

**Figure 1:**
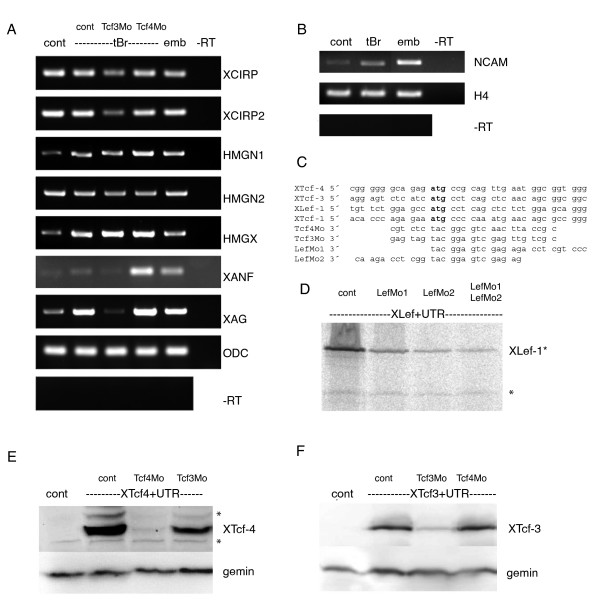
**XCIRP is regulated by XTcf-3, but not by XTcf-4**. (A) RT-PCR on sibling embryos (emb), animal cap explants of naive animal caps (cont), neuralized animal caps (tBr, cont) and neuralized animal caps coinjected with truncated BMP receptor (tBr) and the indicated morpholino (Tcf3Mo: XTcf-3 specific antisense morpholino oligonucleotide, Tcf4Mo: XTcf-4 specific antisense morpholino oligonucleotide) revealed that following XTcf-3 depletion, the expression of XCIRP and XCIRP2, as well as the cement gland specific marker gene XAG is robustly downregulated. The expression of HMGN1, HMGN2 and HMGX is not regulated in a Tcf-subtype specific manner. ODC shows the amplification of the house keeping gene *ornithine decarboxylase*, -RT is the control amplification of not reverse transcribed RNA. (B) RT-PCR on sibling embryos (emb), animal cap explants of naive animal caps (cont) and neuralized animal caps (tBr) demonstrate that the pan-neural marker gene NCAM is induced by injection of 100 pg mRNA encoding for truncated BMP receptor (tBr). H4 shows the amplification of the house keeping gene *histone 4*, -RT is the control amplification of not reverse transcribed RNA. (C) Sequence alignment of the Lef/Tcf specific antisense morpholino oligonucleotides together with the corresponding mRNAs, start codons of the Lef/Tcfs are indicated in bold. Tcf4Mo: XTcf-4 specific antisense morpholino oligonucleotides, Tcf3Mo: XTcf-3 specific antisense morpholino oligonucleotides, LefMo1 and LefMo2: XLef-1 specific antisense morpholino oligonucleotidess. (D-F) Specificity of the Lef/Tcf morpholinos towards their target mRNA, (D) reduction of *in vitro *translated S-35 labelled XLef-1 protein in the presence of the indicated morpholinos, The asterisk marks an unspecific band. (E) reduction of injected C-terminally myc-tagged XTcf-4 by coinjection of ten picomoles Tcf4Mo, but not two picomoles Tcf3Mo. The asterisks indicate unspecific staining. Gemin staining was used as loading control. (F) reduction of injected C-terminally myc-tagged XTcf-3 by coinjection of two picomoles Tcf3Mo, but not ten picomoles Tcf4Mo. Gemin staining was used as loading control.

In order to identify target genes specifically regulated by XTcf-3 in brain development, we subtracted the transcriptome of neuralized animal caps deriving from XTcf-3 morpholino injected embryos from those of XTcf-4 morpholino injected embryos and amplified the remaining cDNAs. Injection of 100 pg RNA encoding a dominant negative BMP receptor was sufficient to induce the pan-neural marker gene NCAM in animal cap tissue (Fig. 1B). Thus, for our subtraction we used a relatively homogenous and defined tissue, simply differing in the presence/absence of XTcf-3 and XTcf-4. Genes regulated by Lef/Tcf transcription factors in general should be eliminated during subtraction and only XTcf-3 specific target genes should be enriched.

Among others (additional file 1), we found as putative XTcf-3 specific target genes most abundant, high mobility group nucleosomal binding protein 1 and 2 (HMGN1 and 2), HMGB3, *Xenopus *anterior gradient (XAG) and cold inducible RNA binding protein (XCIRP and XCIRP2).

**Figure 2:**
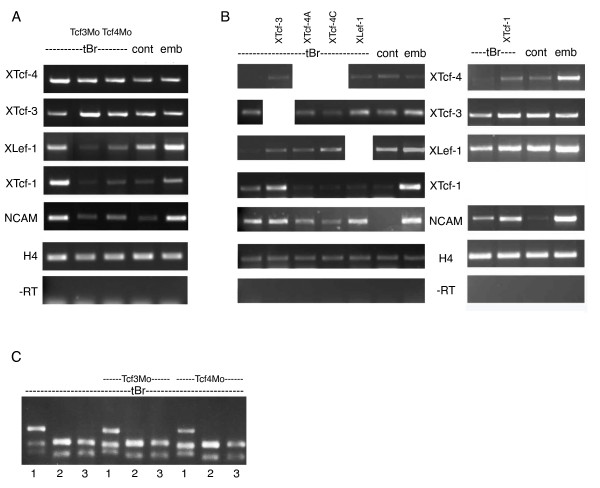
**Regulation of Lef/Tcf expression by Lef/Tcfs in neuralized animal caps**. (A) RT-PCR on sibling embryos (emb), animal cap explants of naive animal caps (cont), neuralized animal caps (tBr) and neuralized animal caps coinjected with truncated BMP receptor (tBr) and the indicated morpholino (Tcf3Mo: XTcf-3 specific antisense morpholino oligonucleotide, Tcf4Mo: XTcf-4 specific antisense morpholino oligonucleotide) revealed that the expression of XLef-1 and XTcf-1 (but not of XTcf-3 and XTcf-4) depends on the presence of XTcf-3 and XTcf-4. NCAM is the amplification of the pan-neural marker gene *neural cell adhesion molecule*, H4 the amplification of the house keeping gene *histone 4*, -RT the control amplification without reverse transcription. (B) Co-injection of 500 pg Lef/Tcf mRNA together with 100 pg tBr indicated complex cross-regulation of Lef/Tcf transcription factors in neuralized animal caps. NCAM is the amplification of the pan-neural marker gene *neural cell adhesion molecule*, H4 the amplification of the house keeping gene *histone 4*, -RT the control amplification without reverse transcription. (C) The pattern of XTcf-4 isoforms remained unchanged following XTcf-3 and XTcf-4 knockdown. Amplicons were digested with isoform-specific restriction enzymes (1: XbaI, 2: RsaI, 3: XbaI + RsaI) and analysed according to [27].

To confirm the target genes, we analyzed *via *RT-PCR whether these genes are down regulated in neuralized animal caps following XTcf-3 depletion (Fig. 1A). Indeed, the expression of XCIRP, XCIRP2 and XAG depended on the presence of XTcf-3, but not on the presence of XTcf-4. Other candidate genes, including the HMG-box proteins HMGN1, HMGN2 and HMGX, however, were not regulated in a Tcf-subtype specific manner. The down regulation of XAG and XCIRP following XTcf-3 depletion, goes ahead with a reduction of the anterior neural marker gene XANF, indicating that the presence XTcf-3 in general is indispensable for anterior neural development. This is further sustained by the finding, that also the pan-neural marker gene NCAM is much stronger downregulated by XTcf-3 depletion than by XTcf-4 depletion (Fig. 2A).

We confirmed the specificity of the morpholinos towards their target-mRNA. Sequence alignment revealed that the subtype specific morpholino oligonucleotides although perfectly matching their target mRNA contain at least 7 mismatches to the mRNA of the other family members (Fig. 1C). In immunoblots we showed, that all three morpholino oligonucleotides suppress translation of their target mRNAs (Fig. 1D, E, F) and that the XTcf-3 specific morpholino has only minor effects on XTcf-4 translation (Fig. 1E) and *vice versa *(Fig. 1F). Consistently, also the RNA-levels of XTcf-3 and XTcf-4 in neuralized animal caps remained unchanged following morpholino injection (Fig. 2A) and the main XTcf-4 isoform in neuralized animal caps is XTcf-4B, regardless whether a morpholino was injected or not (Fig. 2C).

However, analysis of XLef-1 and XTcf-1 in XTcf-3- and 4 depleted neuralized animal caps (Fig. 2A) and in caps overexpressing Lef/Tcfs (Fig. 2B) revealed a complex cross-regulation of these transcription factors. Expression of XTcf-1 and XLef-1 depended on the presence of XTcf-3 and XTcf-4 and overexpression of XTcf-3 induced both, XTcf-1 and XLef-1 (Fig. 2B). XTcf-4 overexpression, however, induced XLef-1 but repressed XTcf-1 (Fig. 2B). The complexity of this cross-regulation is further sustained by the finding that XTcf-3 expression is specifically upregulated following XLef-1 and XTcf-1, but not XTcf-4 mRNA injection, regardless whether an activating (XTcf-4C) or repressing (XTcf-4A) isoform was used. Regulation of the direct wnt target gene XTcf-4 by Lef/Tcfs is seen by the induction of XTcf-4 following XTcf-3, XTcf-1 and XLef-1 overexpression (Fig. 2B).

Thus, a complex cross-regulation of Lef/Tcfs seems to complicate the analysis.

**Figure 3:**
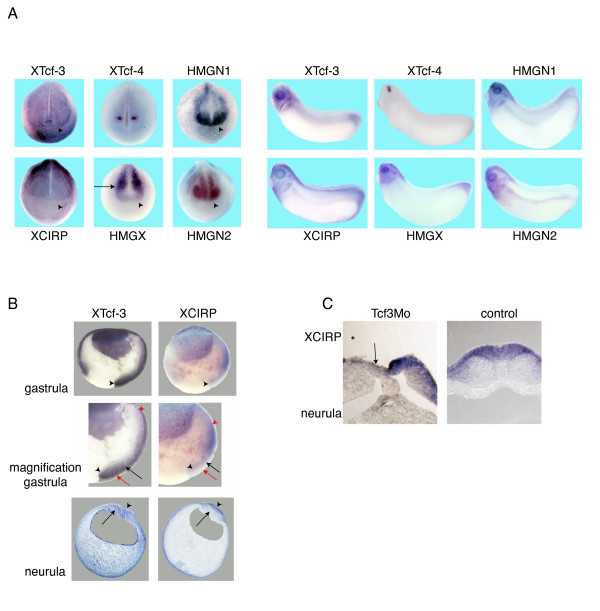
**Coexpression of XTcf-3 and putative XTcf-3 target genes**. *In situ *hybridization reveals synexpression of XTcf-3, HMGN1, HMGN2, HMGX and XCIRP throughout early embryonic development. (A) At neurula stages all five genes are co-expressed and demarcate the anterior border of the neural plate (arrowhead). The main expression of HMGX is found in more posterior neural tissue (arrow) adjacent to the XTcf-4 expression field. The coexpression persists during early embryogenesis and is found in tailbud stages in head structures, including brain, eye, otic vesicle and branchial arches. (B) During gastrulation, XTcf-3 and XCIRP are co-expressed at the dorsal blastopore lip (arrowhead upper panel). The higher magnification reveals that both genes are expressed in the involuting mesoderm (anterior most mesoderm is indicated by a red arrowhead). In the endoderm (black arrowhead) underlying the involuting mesoderm, neither XTcf-3 nor XCIRP are expressed. While XTcf-3 is predominantly localized in the mesoderm (black arrow), the highest expression of XCIRP is found in the ectoderm (red arrow). But still substantial amounts of XCIRP RNA are detected in the mesoderm (black arrow). In anterior transversal sections of stage 18 embryos (lower panel) mRNA of both, XTcf-3 and XCIRP, is located in the neuroectoderm (arrowhead). While XTcf-3 is additionally found in the paraxial mesoderm (arrow), the most prominent XCIRP staining apart from the neural plate is located in the epidermis. (C) A higher magnification of transversal sections shows that following XTcf-3 morpholino injection (Tcf3Mo) XCIRP is strongly reduced in the neural plate (arrow) at the injected side (marked with an asterisk).

If the potential target genes identified in neuralized animal caps are endogenously regulated by XTcf-3, they should be co-expressed with XTcf-3. Indeed, *in situ *hybridization revealed that HMGN1 and 2, HMGX and XCIRP are broadly co-expressed with XTcf-3 at late neurula stages in the neural plate (Fig. 3A). While XTcf-3, HMGN1 and 2 and XCIRP demarcate the anterior border of the neural plate, highest expression of HMGX is found in the prospective midbrain and hindbrain, posterior to the XTcf-4 expression field, but still overlapping with XTcf-3. The co-expression persists throughout development. In tailbud stages, XTcf-3, HMGN1 and 2, HMGX and XCIRP are prominently expressed in the head, including brain, eye, otic vesicles and branchial arches of the neural crest. A closer look-up reveals a partial co-localisation of XCIRP and XTcf-3 at gastrula stages, where both genes are expressed in the involuting mesoderm and at neurula stages, where they are co-localized in the neural plate (Fig. 3B). Thus, XCIRP is expressed throughout early embryogenesis with a broad overlap to XTcf-3 (see also additional file 2).

**Figure 4:**
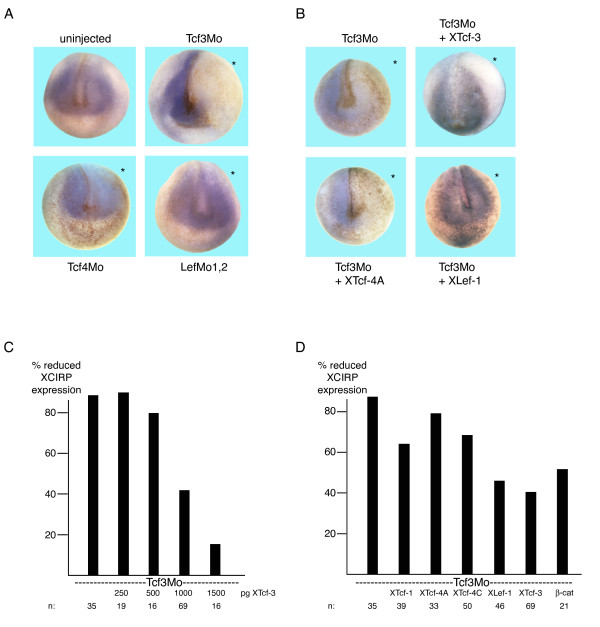
**XCIRP is a Tcf-subtype specific target gene**. (A) Anterior view of *in situ *hybridization reveals that depletion of XTcf-3 by morpholino injection (Tcf3Mo), but not depletion of XTcf-4 (Tcf4Mo) or XLef-1 (LefMo1,2) results in an absence of XCIRP staining at the injected side (asterisk). (B) XCIRP expression in Tcf3Mo injected embryos can be restored by co-injection of XTcf-3 and XLef-1 mRNA. The asterisks mark the injected side. (C, D) The rescue of XCIRP expression by XTcf-3 is dose dependent and Tcf-subtype specific. In (C): the indicated amount of N-terminal myc tagged XTcf-3mRNA was co-injected with two picomoles of Tcf3Mo. In (D): 1000 pg of the indicated Lef/Tcf mRNA or 300 pg β-catenin mRNA were coinjected with two picomoles of Tcf3Mo. Given is the percentage of embryos showing reduced or absent XCIRP expression at the injected side. n: number of analyzed embryos.

Unilateral depletion of XTcf-3 results only in a minor misregulation of HMG-box gene expression. While the expression of HMGN1 was not at all altered by the XTcf-3 morpholino, HMGN2 and HMGX appear expanded at the injected side (additional file 3). The expression of XCIRP in the neural plate, however, clearly depends on the presence of XTcf-3. Following XTcf-3 morpholino injection the expression of XCIRP was robustly reduced at the injected side (Fig. 3C). This regulation was Tcf-subtype specific since depletion of XTcf-4 and XLef-1 did not alter XCIRP expression (Fig. 4A). Co-injection of XTcf-3 mRNA together with the XTcf-3 morpholino restored XCIRP expression in a dose dependent manner (Fig. 4B, C), indicating that XCIRP indeed is regulated by XTcf-3. Accordingly, co-injection of XTcf-1 and XTcf-4A or -C did not restore XCIRP expression while surprisingly, XLef-1 and to a minor extent β-catenin did (Fig. 4B, D). Thus, XCIRP is a Tcf-subtype specific target gene. Its zygotic expression in the neural plate strictly depends on the presence of XTcf-3 and not on XLef-1 and XTcf-4. However, overexpressed XLef-1 can replace endogenous XTcf-3.

**Figure 5:**
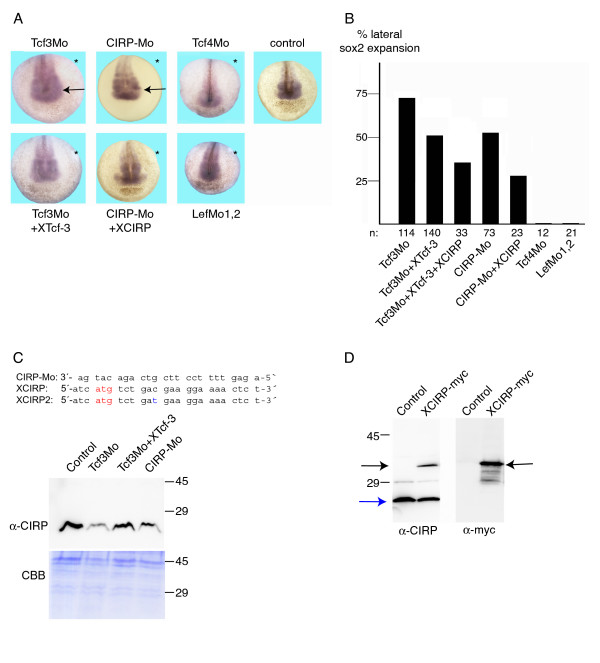
**Depletion of XTcf-3 and XCIRP results in a lateral expansion of sox2**. (A) *In situ *hybridization revealed that both, XTcf-3 and XCIRP are required for proper neural development. The expression of sox2 was laterally expanded (arrow) at the injected side (asterisk). Two picomoles Tcf3Mo, eight picomoles Tcf4Mo or two picomoles CIRP-Mo or two picomoles LefMo1 + two picomoles LefMo2 were coinjected with 4 pg dextrane (to trace the injected side) and 1000 pg XTcf-3 mRNA or 200 pg XCIRP DNA into one blastomere of 2-cell stage embryos. (B) Quantification of the lateral expansion. 71,2% of the Tcf3Mo, 51,5% Tcf3Mo+XTcf-3, 36% Tcf3Mo+XTcf-3+XCIRP, 53% of the CIRP-Mo and 30% of the CIRP-Mo+CIRP and 0% of the Tcf4Mo and LefMo, injected embryos showed a lateral expansion of sox2. n: number of analyzed embryos. (C) The alignment of the XCIRP antisense morpholino oligonucleotide (CIRP-Mo) with XCIRP and XCIRP2 indicates that it is supposed to block both isoforms. The Western Blot demonstrates that the amount of endogenous XCIRP protein is reduced following CIRP-Mo injection. The reduction of XCIRP protein by blocking XTcf-3 translation (Tcf3Mo) can be restored by co-injection of XTcf-3 mRNA (Tcf3Mo + XTcf-3). Two picomoles CIRP-Mo, two picomoles Tcf3Mo and two picomoles Tcf3Mo + 1000 pg XTcf-3 mRNA were injected into both blastomeres of 2-cell stage embryos. RIPA lysates corresponding two 1/3 embryo where separated on a 15% SDS page and either stained with coomassie (CBB) as loading control or transferred to nitrocellulose and stained with anti-CIRP polyclonal antiserum (α-CIRP).(D) The anti-CIRP polyclonal antiserum (α-CIRP), originally directed against XCIRP2 [18] recognizes both, endogenous XCIRP/XCIRP2 (blue arrow) and overexpressed XCIRP (black arrow). Staining with the anti-myc antibody shows the overexpressed myc-tagged XCIRP (black arrow) and some faster migrating proteins, which correspond most likely to degradation products.

**Figure 6:**
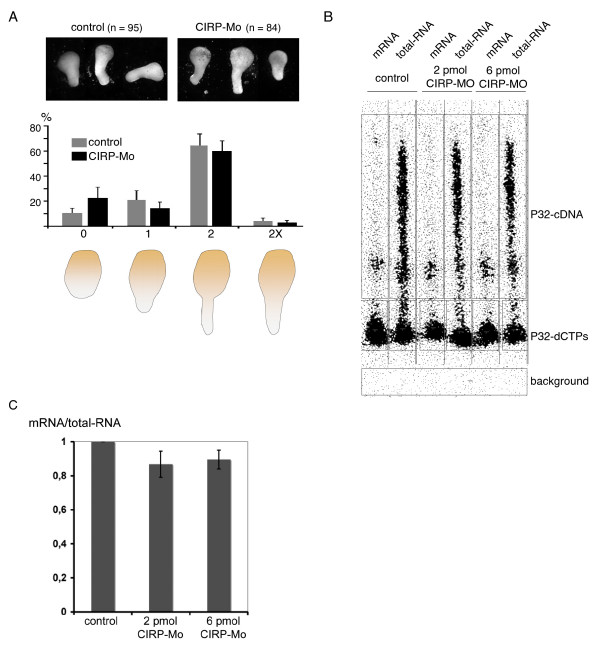
**Depletion of XCIRP causes reduction of mRNA**. (A) XCIRP is not necessary for convergent extension movements. Similar to dorsal animal zone explants of control embryos, more than 60 percent of the explants of CIRP-Mo injected embryos elongate. 0 and 1 indicate non-elongated explants, 2 indicate elongated and restricted explants and 2X elongated, but not constricted explants according to Schambony and Wedlich [27]. (B, C) Depletion of XCIRP results in a reduction of mRNA. Total RNA of stage 16 embryos was reverse transcribed either with oligo dT primer (mRNA) or random hexamer primer (total RNA) in the presence of 60 μCi P32 α-dCTP. (B) Shows an autoradiograph of a representative gel with a smear of labeled cDNA, the free radioactive nucleotides and the background. (C) The signals were quantified by setting the relation of mRNA/total RNA for the control as 1. Shown is the average and standard error for 3 independent experiments.

Apart from the total absence of XCIRP staining at the injected side a severe phenotype following XTcf-3 depletion was an expansion of anterior neural tissue as shown by the expression of HMGN1 and HMGX (additional file 3), and more obviously by the lateral expansion of sox2 (Fig. 5A). Again, this phenotype was specific for XTcf-3 depletion. Depletion of XLef-1 and XTcf-4 did not alter sox2 expression. Co-injection of XTcf-3 mRNA partially restored normal sox2 expression (Fig. 5B). Interestingly, although co-injected XCIRP mRNA did not restore normal sox2 expression in XTcf-3 depleted embryos (data not shown), coinjection of XCIRP mRNA together with XTcf-3 mRNA revealed a better rescue than coinjection of XTcf-3 alone (Fig. 5B). Furthermore, similar to XTcf-3 depletion, also downregulation of XCIRP resulted in a lateral expansion of sox2 expression at the injected side (Fig. 5A, B), suggesting, that XCIRP as XTcf-3 target gene is necessary for proper neural development. Injection of CIRP-Mo and XTcf3Mo resulted in a similar downregulation of XCIRP protein (Fig. 5C). Due to the high similarity of XCIRP and XCIRP2, we assume that the morpholino suppresses the translation of both isoforms. Indeed, the XCIRP specific antibody directed against XCIRP2 [18] also recognized overexpressed XCIRP (Fig. 5D),

The lateral expansion of anterior neural tissue following XCIRP or XTcf-3 depletion could be due to misregulation of morphogenetic movements during neural tube formation, or to alteration in cell fate determination. In whole embryos, we never observed induction or repression of pan-neural, -epidermal or -mesodermal marker genes following morpholino-injections (data not shown), suggesting that the general cell fate decisions are not altered by the morpholinos. Instead, meis3 at the posterior midbrain and hindbrain was expanded and eya1, demarcating the placodal region was shifted laterally at the injected side (additional file 4), most likely as consequence of the lateral expansion of anterior neural tissue. To determine whether convergent extension movements are misregulated following morpholino injection, we analyzed the elongation of Keller open face explants [23] and found that depletion of XCIRP has no effect on these movements (Fig. 6A).

The molecular mechanism of XCIRP, regulating proper development of neural tissue is most likely due to its function as RNA-binding protein. XCIRP is known to stabilize a large number of different mRNAs, including N-cadherin, E-cadherin, β-catenin, cyclin B1, Nek2B, Cytochrome C oxidase peptide VIb and bystin [19,21]. In order to determine, whether a small subset of mRNA or mRNA in general is stabilized by XCIRP, we compared the mRNA/RNA relation of control embryos with XCIRP depleted ones. Indeed, following XCIRP morpholino injection, the mRNA pool was reduced by 10–15% (Fig. 6B, C) indicating that XCIRP as one of the most abundant RNA binding proteins in early *Xenopus *development is involved in stabilizing many different mRNA populations.

Thus, we identified in here the RNA binding protein XCIRP as novel Tcf-subtype specific target gene, which by stabilization of a large pool of mRNAs allows proper anterior neural development.

## Discussion

In a subtractive screen specific for Tcf-subtype specific target genes one of the most frequently candidate genes, *cold inducible RNA binding protein *XCIRP, was found to be regulated by XTcf-3, but not by other Lef/Tcfs. Cement gland specific marker genes, although not co-localized with XTcf-3 in the embryo, have been shown to be repressed by injection of dominant negative XTcf-3 [24]. Consistently, among our XTcf-3 target genes we identified the cement gland specific marker genes XAG and XAG2. Other candidate genes enriched in the subtractive screen are supposed to be expressed rather in ectodermal than in neuroectodermal tissue (α-tubulin, cytokeratine type II, cytokeratine 81 [25,26] or their expression did not strictly depend on the presence of XTcf-3 (HMGN1, HMGN2, HMGX). Interestingly, we did not enrich any "classical" wnt target gene in our screen, which might be explained by their partial redundant roles in transducing wnt/β-catenin signaling or by cross-regulation of their expression. Based on our results, one could suspect, that general wnt/β-catenin target genes are not enriched in the subtractive screen, because both, the XTcf-3 depleted and the XTcf-4 depleted pool also contained less XTcf-1 and XLef-1. Thus, only subtype-specific target genes will be detected, while general wnt/β-catenin target genes are underrepresented.

Although differential activation of wnt/β-catenin target gene promoters by individual Lef/Tcf family members have been reported over the last years [9,24,27-29], most studies dealing with wnt/β-catenin signaling still unite Lef-1, Tcf-1, Tcf-3 and Tcf-4 as Lef/Tcfs and implicate that they are exchangeable. Thus far, non-sequence specific DNA binding and recruitment of p300 by the Tcfs E-tail [30,31] and epigenetic modifications of wnt target gene promoters [29] provide the best explanations for subtype specific target gene regulation. Recently, it has been shown in *Drosophila*, that pangolin/armadillo also promotes target gene repression by binding to DNA sequences different to the "classical" Lef/Tcf binding side [32]. Whether a similar mechanism also regulates Tcf-subtype specificity in vertebrates remains elusive.

With XCIRP, we describe for the first time a novel Lef/Tcf target gene, which is endogenously regulated in a Tcf-subtype specific manner. XTcf-3 but not XTcf-4 and XLef-1 is required for the XCIRP expression in the neural plate.

Furthermore, XCIRP expression can be restored by XTcf-3 but not by XTcf-1 and XTcf-4, indicating that Lef/Tcfs are not exchangeable. However, the fact that XLef-1 can restore XCIRP expression in a similar extent as XTcf-3 revealed some redundant functions of XTcf-3 and XLef-1. This redundant function is somehow unexpected, because we recently could show that XTcf-3 in general behaves as a repressor and XLef-1 as an activator [27,28]. Keeping in mind the redundant functions of murine Lef/Tcfs revealed by loss of function studies and double knockouts in mice [7,8] it is surprising, that we could detect such specific regulation of XCIRP expression by individual Lef/Tcfs, even more, since it has been shown in different contexts that the expression of Lef/Tcf transcription factors is regulated by Lef/Tcfs (this study, 33,34,35). However, this complex cross-regulation provides an explanation for the unexpected reconstitution of XCIRP expression following XLef-1 injection in XTcf-3 depleted embryos: Because overexpression of XLef-1 upregulates XTcf-3, the recovery of XCIRP might be a secondary effect and still due to a subtype-specific regulation by XTcf-3. ChIP with XTcf-subtype specific antibodies will help to define whether endogenous XCIRP, indeed, is directly regulated by XTcf-3 and not by XLef-1.

Lef/Tcf transcription factors as transducer of the wnt/β-catenin signaling pathway have been reported to regulate anterior posterior patterning of the CNS. While activation of wnt/β-catenin signaling in general posteriorizes the CNS [12], wnt-2b/XTcf-4 regulates the development of the midbrain [6]. Here we show that XTcf-3 is required for proper development of the neural plate. Depletion of XTcf-3 results in a lateral expansion of the pan-neural marker gene sox2, the midbrain specific marker gene Meis3 and to a shift of the placodal marker gene eya1. A similar broadening of the anterior neural plate was observed following injection of dominant negative XTcf-3 [24]. Thus, inhibition of wnt/β-catenin signaling is required for neural induction and injection of dn XTcf-3 is thought to mimick the effect of endogenous wnt inhibitors. In contrast to the dominant negative XTcf-3 in Heeg-Truesdells study [24], the XTcf-3 morpholino did not result in neural induction. This might be due to the fact that the N-terminal deletion mutant competes with all Lef/Tcfs, thus is not strictly specific for XTcf-3.

A similar broadening of the neuroectoderm was also observed following depletion of the transcription factors zic-1 [36] and six1 [37]. In contrast to the depletion of zic-1 and six1, the broadening of the anterior neural tissue following depletion of XTcf-3 did not go ahead with a reduction of placodal marker genes, thus, did not affect the organization of the neural plate border. Although wnt/β-catenin signaling is involved in the induction of cranial placodes [38], the expression of the placodal marker gene eya1 seems to be unaffected by depletion of XTcf-3. This opens up the question, how XTcf-3 can lead to a broadening of the neural plate without affecting the integrity of the adjacent placodal region. We provide evidence, that stabilization of a large pool of mRNAs by the XTcf-3 target XCIRP might be an explanation.

Depletion of the XTcf-3 target gene XCIRP resulted in a similar lateral expansion of neural tissue, indicating that XCIRP is an important downstream component of XTcf-3 for neural development. One obvious mechanism for this broadening would be a failure in convergent extension movements, which might be caused by a destabilization of the mRNA of cell adhesion molecules including C- and E-Cadherin, β-Catenin, and α-Catenin [21]. According to our dorsal marginal zone explants, we can exclude an effect on convergent extension movements, because injection of the CIRP morpholino did not result in an inhibition of elongation or constriction of the explants. The molecular mechanism of XCIRP action during early embryonic development is most likely due to a stabilization of mRNA. CIRP has been shown to bind to and stabilize many different mRNAs, including those encoding for cell adhesion molecules and cell proliferation regulators [19,21]. Thus, CIRP is supposed to act as a general mRNA binding protein, which regulates the stability of a large pool of different mRNAs. Consistently, we can show that the total mRNA pool is decreased following XCIRP morpholino injection, indicating that apart the thus far identified XCIRP target mRNAs a big pool of additional mRNAs is regulated by XCIRP. Thus, we conclude that the XTcf-3 target gene XCIRP regulates the correct positioning of the neural border by regulating the stability of many different mRNAs. We think that deciphering the identity of the XCIRP regulated mRNA pool will give enormous progress in understanding the mechanisms underlying early neural development and the positioning of the neuroectoderm/ectoderm border. Furthermore, the specific effect of XCIRP depletion on neural development together with the distinct expression domains of different RNA binding proteins including XCIRP, SEB4 [39], nrp-1 [40] and elav-like proteins [41] might reflect a general mechanism in development: stabilization of a distinct cell fate by regulating the stability (or translation) of a broad variety of mRNAs in a tissue specific manner.

## Conclusion

In a screen for Tcf-subtype specific target genes we identified cold inducible RNA binding protein XCIRP as novel XTcf-3 specific target gene. Both, XTcf-3 and XCIRP are required for proper anterior neural development. We provide evidence, that RNA stabilization by the XTcf-3 specific target gene XCIRP is essential for anterior neural development.

## Methods

### Plasmids, constructs, *in vitro *transcription

Capped mRNAs were transcribed from linearized DNA templates using mMESSAGE mMACHINE (Ambion). Digoxigenin labeled antisense probes for *in situ *hybridization were synthesized with DIG RNA labeling kit (Roche). Probes for *in situ *hybridization are described elsewhere: sox2 [15], Tcf-4 [5], HMGN1 and HMGN2 [42], HMGX [43], Meis3 and eya1 [44]. The open reading frame of XCIRP was amplified using the following primers: XCIRPstart: 5'-tcagaattcaatgtctgacgaaggaaaact-3' and XCIRPstop 5'-cttctcgagttactcgtgtgtagcatagctg-3' and sub-cloned into pGEMT for creating labeled antisense RNA and into pCS2 for synthesizing myc tagged mRNA. cDNA corresponding to aa 63–328 of XTcf-3 [45] was inserted into pGEMT for creating labeled antisense RNA. The following antisense morpholino oligonucleotides (Gene Tools, LaJolla) were used: XTcf-3 (Tcf3Mo, MO#-Tcf7l1): 5'-cgctgttgagctgaggcatgatgag-3' (directed against BC077764); XTcf-4 (Tcf4Mo, MO#-tcf4): 5'-cgccattcaactgcggcatctctgc-3' (directed against BC106476), XLef-1 LefMo1, MO#-LEF1): 5'-tacggagtcgagagacctcgtccc-3' and (LefMo2, MO#-LEF1): 5'-caagacctcggtacggagtcgagag-3' (directed against NM_001088655 und AC151234) and XCIRP (CIRP-Mo, MO#-CIRBP): 5'-agtacagactgcttccttttgaga-3' (directed against XCIRP NM_00108600, XCIRP1 AF278702 and XCIRP2 NM_001086325). The concentrations injected are given in the figure legends and were tested to be non-toxic.

### Selective subtractive screen

Eggs from HCG treated females were fertilized by standard methods and staged according to [46]. For animal cap explants eight picomoles of XTcf-3 and XTcf-4 morpholino where co-injected with 100 pg mRNA encoding for a truncated version of the BMP receptor (tBr) into both blastomeres of 2-cell stage embryos. At late blastula, animal caps where explanted and cultivated until sibling embryos reached stage 16. The transcriptome of Tcf-3 depleted neuralized animal caps was subtracted from the transcriptome of Tcf-4 depleted ones using the "PCR-Select cDNA Subtraction Kit" (BD Biosciences, Heidelberg, Germany) according to manufacturers description.

### Embryo treatment and *in situ *hybridization

For marker gene analysis, the indicated morpholinos where co-injected into one blastomere of *Xenopus *2-cell stage embryos with 4 pg dextrane or 100 pg mRNA encoding for myc-tagged GFP as lineage tracer. At neurula stages, embryos were fixed in MEMFA (0.1 M MOPS pH 7.2, 2 mM EGTA, 1 mM MgSO_4_, 3.7% formaldehyde). For vibratome sections, fixed embryos were embedded in 3% agarose and sectioned using a Leica VT 1000S vibratome. Whole-mount *in situ *hybridization was performed according to previously described procedures [47]. Localization of mRNA was visualized using anti-digoxigenin antibodies conjugated to alkaline phosphatase, followed by incubation with nitro blue tetrazolium (NBT) and 5-bromo 4-chloro 3-indolyl phosphate (BCIP). Images were captured on a Leica MZFLIII microscope using a digital camera (Qimaging) and Improvision software (Openlab).

For analyzing convergent extension movements, dorsal marginal zone explants were cut at blastula stages, cultivated until siblings reached stage 16 and analyzed according to [23].

### Immunoblotting

RIPA lysates corresponding to one half embryo were separated by SDS-PAGE and transferred onto nitrocellulose. After blocking, the membrane was incubated with α-myc (9E10) or α-CIRP antibody (kindly provided by Ken Matsumoto) and peroxidase-coupled secondary antibody. Proteins were visualized using ECL-Plus Western Blotting Detection System (Amersham). As loading control, blots were incubated with α-Gemin antiserum kindly provided by U. Fischer, or a parallel Gel was stained with Coomassie brillant blue.

### Marker gene analysis

For marker gene analysis by RT-PCR, total RNA was extracted from whole embryos or animal caps, reverse transcribed using random hexamer primers and MMLV reverse transcriptase. For amplification the following primers were used. Histone 4: 5'-cgggataacattcagggtatcact-3' and 5'-atccatggcggtaactgtcttctt-3'; XCIRP: 5'-gctgatcaggcggggccacc-3' and 5'-gcacccaggctctgtcctgc-3'; XCIRP2: 5'-ccattcaggctgatcagg-3' and 5'-ctggagagagacgaacac-3'; XAG: 5'-gagttgcttctctggca-3' and 5'-ctgactgtccgatcagac-3'; XANF: 5'-actgacctacaagagagaa-3' and 5'-agtgcatcattgttccacag-3'; ODC: 5'-tggcagcagtacagacagca-3' and 5'-gatgggctggatcgtatcct-3'; XTcf-1: 5'-ctcggctcacatgga agatg-3' and 5'-cagatcgacaggaaggtg tc-3'; XLef-1: 5'-ggagacctcgcagatatcaa-3' and 5'-ccagccc aatggtggtgtcat-3'; XTcf-3: 5'-catggcagcatgctcgactc-3' and 5'-ctggtcactagagaaagggg-3'; XTcf-4: 5'-ctcacgccgctcattacctacagcaac-3' and 5'-catgtacagcatgaacgcgtttagggg-3'; HMGN1: 5'-ggct cctgcttctgatggag-3' and 5'-ccagtatcaatctctgcctg-3'; HMGN2: 5'-ggggacacaaagtgaagc-3' and 5'-cacagtcgaagtaccgcc-3' and HMGX: 5'-cgtccccagataaagagcgag-3' and 5'-gctgcactcgagttcac attc-3'. NCAM: 5'-ggaatcaagcggtacaga-3' and 5'-cacagttccaccaaatgc-3'. The XTcf-4 isoforms were determined by digesting the amplicons with RsaI and XbaI as previously described [27].

### mRNA/total RNA ratio

For analyzing the mRNA/total RNA ratio, 1 μg RNA of stage 18 embryos was reverse transcribed with MMLV reverse transcriptase and random hexamer primers (total RNA) or oligo-dT primers (mRNA) in the presence of 5 μCi [^32^P]-α-dCTP (3000 Ci/mmol). Radioactive transcripts were separated from unincorporated [^32^P]-α-dCTP nucleotides by denaturating polyacrylamide gel electrophoresis. Quantity of transcripts was determined via intensity of radioactive signals (Canberra Packard) and normalized to total radioactivity.

## Authors' contributions

SvV carried out most of the injection experiments and embryo analyses. DF carried out injection experiments and embryo analyses. MK performed the subtractive screen. DW participated in the design and coordination of the study and helped to draft the manuscript. DG conceived of the study, participated in embryo analyses and drafted the manuscript. All authors read and approved the final manuscript.

## Supplementary Material

Additional File 1 Table of putative XTcf-3 target genes. Click here for file

Additional File 2 **Expression of XCIRP during early embryogenesis**. (A) Spatial expression of XCIRP during early development as revealed by *in situ *hybridization. The arrows indicate high expression of XCIRP in neural tissue. Arrowheads point to the expression in the branchial arches. The red arrow indicates staining of the pronephros. The open reading frame of XCIRP was amplified using the following primers: XCIRPstart: 5'-tcagaattcaatgtctgacgaaggaaaact-3' and XCIRPstop 5'-cttctcgagttactcgtgtgtagcatagctg-3' and sub-cloned into pGEMT for creating labeled antisense RNA. (B) Temporal expression of XCIRP and XCIRP2 as revealed by RT-PCR. H4 indicates the amplification of the house keeping gene histone 4, -RT the amplification of H4 in samples, which have not been reverse transcribed. The following primer pairs were used: histone 4: 5'-cgggataacattcagggtatcact-3' and 5'-atccatggcggtaa ctgtcttctt-3'; XCIRP: 5'-gctgatcaggcggggccacc-3' and 5'-gcacccaggctctgtcctgc-3'; XCIRP2: 5'-ccattcaggctgatcagg-3' and 5'-ctggagagagacgaacac-3'. Click here for file

Additional File 3 **Depletion of XTcf-3 has minor effects on the expression of HMG-box genes**. Depletion of XTcf-3 by injection of two picomoles morpholino (Tcf3Mo): 5'-cgctgttgagctgaggcatgatgag-3' (directed against BC077764) into one blastomere of 2-cell stage embryos (the injected side is indicated by an asterisk) has only minor effects on the expression of HMGN1, HMGN2 and HMGX. While HMGN2 and HMGX are laterally expanded (arrow), HMGN1 remains unchanged. Probes for *in situ *hybridization are as described: HMGN1 and HMGN2 [1], HMGX [2]. Click here for file

Additional File 4 **Depletion of XTcf-3 and XCIRP results in a lateral shift of the neuroectodermal border**. (A) Lateral expansion (arrows) of Meis3, (B) lateral shift (arrows) of eya1 expression following unilateral injection of two picomoles XTcf-3 morpholino (Tcf3Mo), XTcf-3 morpholino together with 1000 pg XTcf-3 mRNA (Tcf3Mo + XTcf-3) or two picomoles XCIRP-morpholino (CIRP-Mo: 5'-agtacagactgcttccttttgaga-3'). The asterisks mark the injected side. The quantification gives the percentage of embryos showing lateral expansion of Meis3 (A) or shift of eya1 (B). N gives the number of analyzed embryos. Probes for *in situ *hybridization are as described [3]. Click here for file
