# Supplementary material for: Cold-inducible RNA binding protein (CIRP), a novel XTcf-3 specific target gene regulates neural development in Xenopus
Source: BMC Dev Biol. 2008 Aug 7;8:77. doi: 10.1186/1471-213X-8-77 (PMC2527318; doi:10.1186/1471-213X-8-77)
Supplement: Additional File 1 — Table of putative XTcf-3 target genes. [file 1471-213X-8-77-S1.pdf]

Additional file 1 –table of putative XTcf-3 target genes

Clone	Frequency	Accession number
α-Tubulin	23	BC106380
Similar to α-Tubulin	2	NM_001086054
Cytokeratine type II	19	NM_001087056
Cytokeratine 81	6	NM_001088963
XAG2	12	NM_001088200
XAG	4	NM_001086198
Similar to XAG	3	NM_001086251
CGS	2	BC128956
XCIRP	11	NM_001086600
XCIRP2	1	NM_001086325
Mitochondrial DNA	9	M10217
HMGN2	7	NM_001088261
HMGN1	2	NM_001087294
HMGB3	1	NM_001095694
XEEL	5	NM_001089101
Hsc70	3	NM_001086599
Gfpt1	2	NM_001087014
