# Supplementary figures and images for: Cold-inducible RNA binding protein (CIRP), a novel XTcf-3 specific target gene regulates neural development in Xenopus
Source: BMC Dev Biol. 2008 Aug 7;8:77. doi: 10.1186/1471-213X-8-77 (PMC2527318; doi:10.1186/1471-213X-8-77)

A

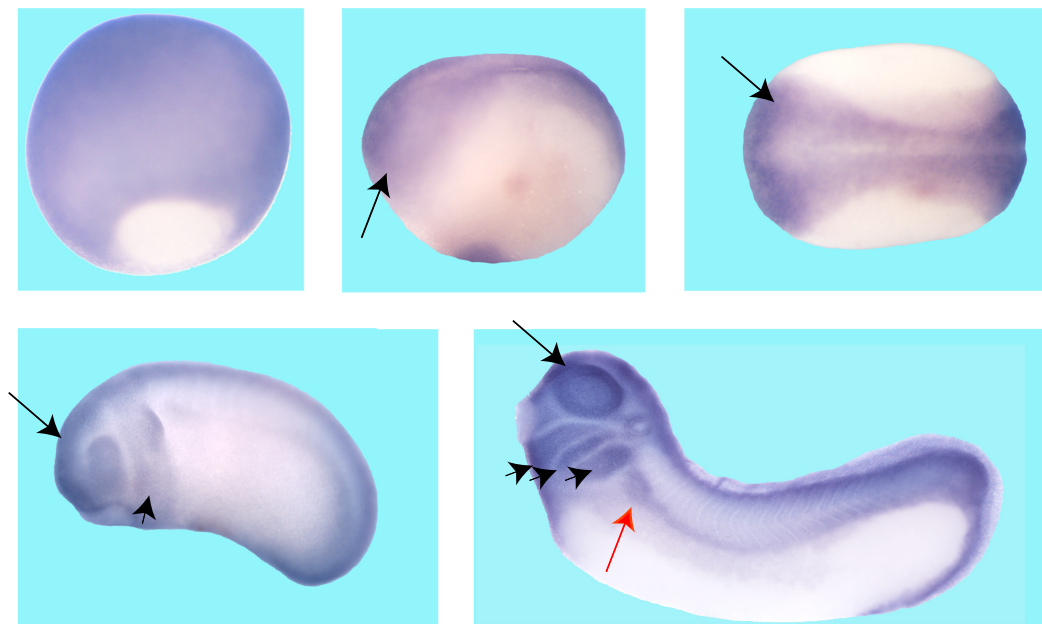

B

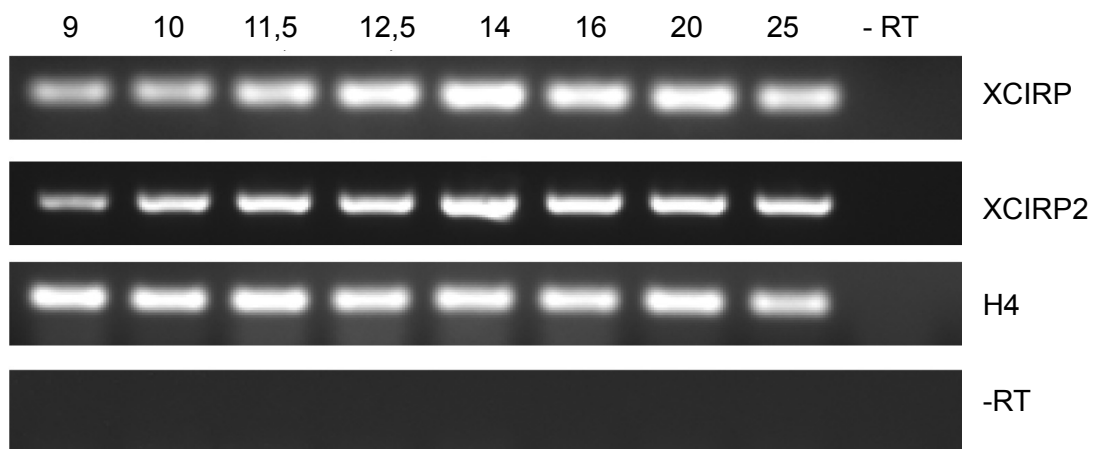

Supplement: Additional File 2 — Expression of XCIRP during early embryogenesis. (A) Spatial expression of XCIRP during early development as revealed by in situ hybridization. The arrows indicate high expression of XCIRP in neural tissue. Arrowheads point to the expression in the branchial arches. The red arrow indicates staining of the pronephros. The open reading frame of XCIRP was amplified using the following primers: XCIRPstart: 5'-tcagaattcaatgtctgacgaaggaaaact-3' and XCIRPstop 5'-cttctcgagttactcgtgtgtagcatagctg-3' and sub-cloned into pGEMT for creating labeled antisense RNA. (B) Temporal expression of XCIRP and XCIRP2 as revealed by RT-PCR. H4 indicates the amplification of the house keeping gene histone 4, -RT the amplification of H4 in samples, which have not been reverse transcribed. The following primer pairs were used: histone 4: 5'-cgggataacattcagggtatcact-3' and 5'-atccatggcggtaa ctgtcttctt-3'; XCIRP: 5'-gctgatcaggcggggccacc-3' and 5'-gcacccaggctctgtcctgc-3'; XCIRP2: 5'-ccattcaggctgatcagg-3' and 5'-ctggagagagacgaacac-3'. [file 1471-213X-8-77-S2.pdf]

# HMGN2

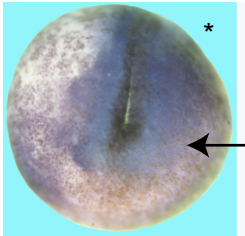

# HMGN1

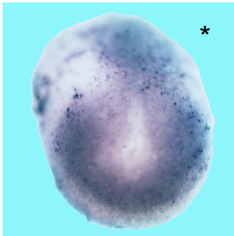

# HMGX

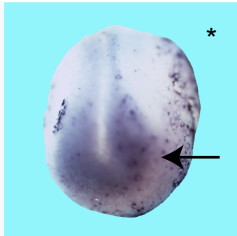

Supplement: Additional File 3 — Depletion of XTcf-3 has minor effects on the expression of HMG-box genes. Depletion of XTcf-3 by injection of two picomoles morpholino (Tcf3Mo): 5'-cgctgttgagctgaggcatgatgag-3' (directed against BC077764) into one blastomere of 2-cell stage embryos (the injected side is indicated by an asterisk) has only minor effects on the expression of HMGN1, HMGN2 and HMGX. While HMGN2 and HMGX are laterally expanded (arrow), HMGN1 remains unchanged. Probes for in situ hybridization are as described: HMGN1 and HMGN2 [1], HMGX [2]. [file 1471-213X-8-77-S3.pdf]

A

3-Mo

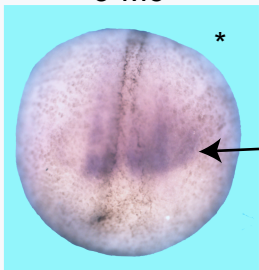Tcf3Mo  
+XTcf-3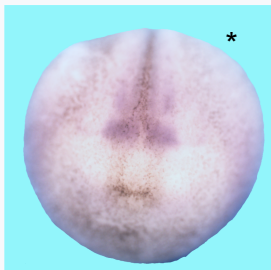

CIRP-Mo

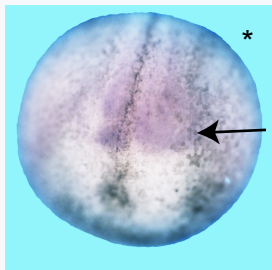% lateral  
Meis expansion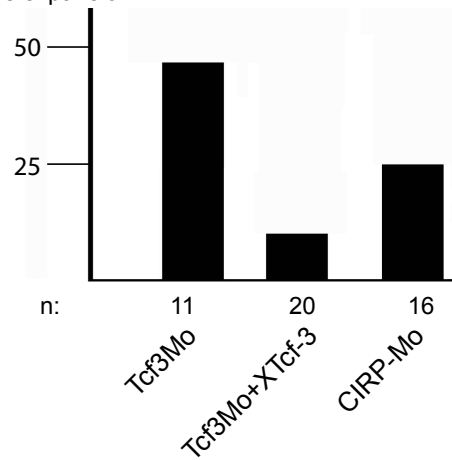

B

3-Mo

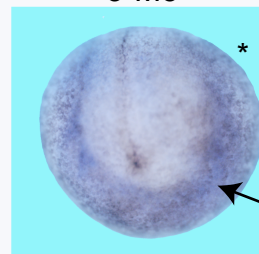Tcf3Mo  
+XTcf-3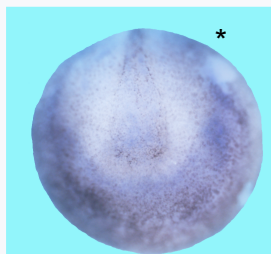

CIRP-Mo

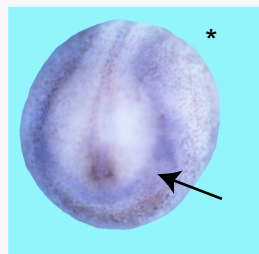% lateral  
eye expansion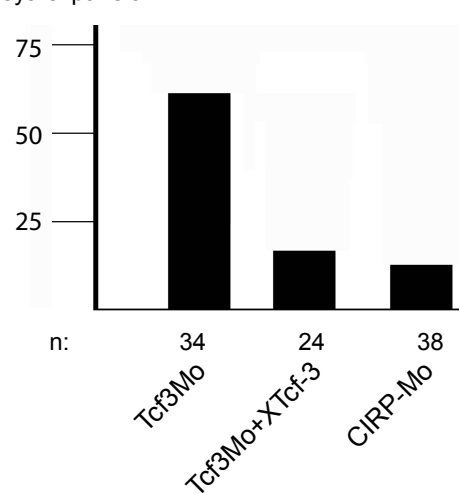

Supplement: Additional File 4 — Depletion of XTcf-3 and XCIRP results in a lateral shift of the neuroectodermal border. (A) Lateral expansion (arrows) of Meis3, (B) lateral shift (arrows) of eya1 expression following unilateral injection of two picomoles XTcf-3 morpholino (Tcf3Mo), XTcf-3 morpholino together with 1000 pg XTcf-3 mRNA (Tcf3Mo + XTcf-3) or two picomoles XCIRP-morpholino (CIRP-Mo: 5'-agtacagactgcttccttttgaga-3'). The asterisks mark the injected side. The quantification gives the percentage of embryos showing lateral expansion of Meis3 (A) or shift of eya1 (B). N gives the number of analyzed embryos. Probes for in situ hybridization are as described [3]. [file 1471-213X-8-77-S4.pdf]
